# Effect of Sugarcane Polyphenol Extract on α-Amylase Inhibition and Mechanism Exploration

**DOI:** 10.3390/foods14132174

**Published:** 2025-06-21

**Authors:** Yumei Wang, Jiulong An, Shenghong Yao, Chengfeng Zhang, Yanv Zhou, Lu Li, He Li

**Affiliations:** 1 Key Laboratory of Geriatric Nutrition and Health, Beijing Technology and Business University, Ministry of Education, Beijing 100048, China; wym110809@126.com (Y.W.); anjiulong0322@163.com (J.A.); 2431031006@st.btbu.edu.cn (S.Y.); lil@btbu.edu.cn (L.L.); 2 The Product Makers Co., Ltd., Shanghai 200444, China; eric.zhang@theproductmakers.cn (C.Z.); joanna.zhou@theproductmakers.cn (Y.Z.)

**Keywords:** sugarcane polyphenols, proanthocyanidin-B1, chlorogenic acid, α-amylase inhibition, stability analysis, molecular dynamics

## Abstract

Although α-amylase is crucial for postprandial glucose control, existing inhibitors present various side effects, necessitating the exploration of natural alternatives. The ability of sugarcane polyphenol (SP) to inhibit α-amylase remains unclear. This study assessed the inhibitory activity of SP via in vitro assays, circular dichroism (CD), fluorescence quenching, and stability analysis, while the mechanism of action was elucidated using molecular docking and molecular dynamics (MD). The results showed that the IC_50_ of the SP was 0.841 ± 0.029 mg/mL, with proanthocyanidin-B1 (PC-B1) presenting the most potent effect (IC_50_ = 0.504 ± 0.019 mg/mL). CD and barycentric mean (BCM) analysis indicated that the complexes might limit substrate binding. The mechanistic assessment showed that the polyphenols bonded to the active enzyme pockets to form stable complexes with reduced key residue fluctuations. In conclusion, SP, especially PC-B1, effectively inhibited α-amylase activity via structural regulation and molecular interactions, providing a theoretical basis for developing natural hypoglycemic agents.

## 1. Introduction

Sugarcane is a vital global source of sugar and biofuel, with an annual worldwide production of 1.9 billion tons between 2020 and 2022 [1], approximately 92% of this production is used for sugar manufacturing [2]. Molasses, a by-product of sugar processing, is rich in soluble sugars and bioactive compounds and is widely utilized in fermentation and food processing. Recent studies have revealed that molasses is rich in phenolic compounds [3], which exhibit various beneficial biological activities, including antioxidant properties, anti-inflammatory capabilities, lipid regulation, and the potential to modulate neurological diseases. Therefore, an extensive investigation into the biological activities of the phenolic compounds in sugarcane molasses can enhance the comprehensive utilization of sugarcane resources, providing a solid scientific basis for the tailored application of sugarcane polyphenol (SP).

SP refers to powdered substances derived from sugarcane molasses using water and ethanol as extraction solvents, followed by filtration, vacuum concentration, ion exchange, and other processes [4]. Several in vitro and in vivo studies have shown that SP may improve intestinal flora, exhibit anti-inflammatory and neuroprotective properties, promote insulin secretion from dysfunctional β-cells, and ameliorate UV-induced skin aging [4,5,6,7]. However, the ability of SP to inhibit α-amylase, as well as the related mechanism of action, remains unclear.

Starchy foods represent a significant human energy source and can rapidly increase postprandial blood glucose levels after digestion. The starch is hydrolyzed into oligosaccharides (e.g., maltose, maltotriose, and dextrin) by salivary and pancreatic α-amylase, followed by further metabolism into glucose. As a crucial enzyme that catalyzes starch digestion and influences glucose release, α-amylase is considered a primary target for postprandial blood glucose control [8]. Prolonged ingestion of commonly used digestive enzyme inhibitors, such as acarbose (ACA) and voglibose, may cause side effects, including bloating and diarrhea. Contrarily, natural polyphenols have attracted considerable attention as potential amylase inhibitors due to their favorable safety profile and broad availability. Studies have demonstrated that the polyphenols in fruits, grains, and tea can effectively inhibit α-amylase activity [9,10,11]. However, the ability of SP to inhibit α-amylase and the related action mechanism remains unclear. Therefore, exploring the inhibitory mechanism of SP and its main components on α-amylase can provide a theoretical foundation for developing targeted sugar-control functional products.

This study aimed to identify phenolic compounds that effectively restrict α-amylase and to reveal their inhibitory mechanisms. The primary components of SP were analyzed using high-performance liquid chromatography–tandem mass spectrometry (HPLC-MS/MS). The repressive effect of various phenolic compounds on α-amylase was investigated via in vitro inhibition assays, circular dichroism (CD), fluorescence quenching, and conformational stability analyses. The mechanisms by which the main components of SP inhibit amylase were elucidated using molecular docking and molecular dynamics (MD). This research is significant for identifying practical amylase-inhibiting components in sugarcane by-products, enhancing the comprehensive utilization of sugarcane resources, and expanding the availability of natural amylase-inhibiting active substances in natural products.

## 2. Materials and Methods

### 2.1. Materials

SP is a brown powder with a total phenol content of 223.69 ± 5.48 mg gallic acid equivalents/g. According to Ji et al., SP is a novel plant extract prepared from sugarcane molasses using a patented hydrophobic resin technology [4]. Briefly, after diluting sugarcane molasses to 20 Brix, 500 g of wet heavy ion exchange resin composite adsorbent was added to 1 L of raw liquid under a temperature of 20–25 °C, and specific binding of the hydrophobic components was achieved by constant stirring. After collecting the resin by vacuum filtration, the resin was washed twice with 1 L of deionized water to remove impurities, followed by elution of the target polyphenol fraction in three fractions using 70% ethanol. After vacuuming and removing ethanol from the combined eluent, the product was prepared by lyophilization or spray drying, resulting in brown powders with good flowability and a water content of 2–4%. The chlorogenic acid (CGA) (HPLC purity > 99%), rutin (RUT) (HPLC purity > 99%), p-coumaric acid (p-CA) (HPLC purity > 99%), ACA, and α-amylase were purchased from Yuanye Biotechnology Co., Ltd. (Shanghai, China), while Refensi Biotechnology Co., Ltd. (Chengdu, China) supplied the proanthocyanidin-B1 (PC-B1) (HPLC purity ≥ 98%). The dimethyl sulfoxide was obtained from Sigma-Aldrich Chemical Co., Ltd. (Milwaukee, WI, USA). The soluble starch (potato starch, straight chain starch: branched chain starch = 2:8) was purchased from Fuchen Chemical Reagent Co., Ltd. (Tianjin, China).

### 2.2. HPLC-MS/MS Profiling of the SP

HPLC-MS/MS was employed to analyze the SP, using Agilent 1290 (Agilent, Santa Clara, CA, USA) and Q Exactive Plus Orbitrap systems (Thermo Fisher Scientific, Waltham, MA, USA), as well as a ChromCore 120 C18 column (NanoChrom, Suzhou, China) (150 mm × 2.1 mm, 1.8 μm), maintained at 40 °C. Mobile phase A consisted of 0.1% formic acid passed, while mobile phase B comprised 100% CAN. Both mobile phases were filtered through a 0.5 μm membrane. The elution gradient included 5–70% B for 0–10 min, 70–100% B for 10–17 min, 100–100% B for 17–18 min, 100–5% B for 18–19 min, and 5–5% B for 19–21 min. The other parameters included a flow rate of 0.3 mL/min and an injection volume of 10 μL. The peak information obtained from the MS data after pre-processing, such as peak extraction, de-noising, de-convolution, and peak alignment, was compared with the database for phenol identification.

### 2.3. α-Amylase Inhibition Assay

The α-amylase activity was determined using a previously delineated method [12]. The inhibitory activity of different SP, CGA, p-CA, PC-B1, and RUT concentrations on α-amylase was determined, with ACA as a positive control. First, 100 μL of the different sample solution concentrations were mixed with 50 μL, 3 U/mL of the α-amylase solution, and incubated at 37 °C for 10 min, after which 100 μL of a pasteurized starch solution (0.04% mass fraction) was added. After 30 min at 37 °C, the reaction was terminated by adding 100 μL of an iodine dilution, and the absorbance was measured at 660 nm using an Infinite M200 Pro Multimode Microplate Reader (Tecan, Mannedorf, Switzerland). The inhibition rate of each substance against α-amylase was calculated using the following formula and expressed as the half inhibitory concentration (IC_50_) value:

$$\mathrm{Inhibition}(\%)=(1-\frac{\left(A1-A2\right)}{\left(A3-A4\right)})*100\%$$

A1 denotes the reaction system without α-amylase (blank 1), A2 represents the reaction system containing α-amylase and an inhibitor, A3 is the reaction system without α-amylase and an inhibitor (blank 2), and A4 signifies the reaction system without an inhibitor.

### 2.4. CD Measurements

Here, 0.6 mg/mL α-amylase was mixed with 0.04 mg/mL of the different substances at a volume ratio of 1:1 and incubated at 37 °C for 5 min. The CD spectral data of the α-amylase with and without phenolics were recorded in a wavelength range of 190–260 nm using a MOS-500 CD spectropolarimeter (Biologic, Seyssinet-Pariset, France and analyzed using CDNN 2.1 software.

### 2.5. Fluorescence Quenching

Fluorescence quenching was performed using a previously described method [13]. An α-amylase solution was mixed with sample solutions at concentrations of 0 μg/mL, 5 μg/mL, 10 μg/mL, 20 μg/mL, 40 μg/mL, and 80 μg/mL at a 1:1 volume ratio, followed by incubation at 37 °C for 5 min. For the control group, the same volume of buffer was used instead of phenolic compounds. A fluorescence spectrofluorometer (FS5, Edinburgh Corporation, Southampton, UK) was employed to measure the fluorescence of the α-amylase at an excitation wavelength of 290 nm and an emission wavelength range of 300–500 nm. The amylase fluorescence spectrum was obtained by subtracting the blank spectrum (the spectra corresponding to the phenolic compound solution and buffer) from the emission spectrum.

### 2.6. Stability Analysis

The α-amylase stability in various phenolic compounds was examined using a previously described method [14]. Denaturation and aggregation temperature modes were employed to assess the impact of phenolic compounds on α-amylase stability using a protein stabilizer unit (Unit, Unchained, Pleasanton, CA, USA). Stability curves were established for various α-amylase concentrations in preliminary experiments. The α-amylase was incubated with 0.2 mg/mL of the phenolic compounds or the positive control at 37 °C for 30 min, after which 9 μL of the solution was pipetted into the sample cell. The temperature was linearly increased from 20 °C to 90 °C at a rate of 0.5 °C/min. Lasers were used to continuously monitor the fluorescence signals of the α-amylase at 266 nm and 473 nm, acquiring the emission spectra from 250 nm to 700 nm. The experimental results were analyzed using the Uncle Analysis V4.01 software.

### 2.7. Molecular Docking

The potential phenolic and α-amylase binding sites were predicted via molecular docking. The α-amylase crystal structure (PDB ID: 1PIF) was obtained from the Protein Data Bank (https://www.rcsb.org/, accessed on 9 April 2024). For docking preparation, a pdbqt file of the receptor was obtained through the dehydration hydrogenation of the α-amylase crystal structure using the AutoDock Vina software 1.5.7. The small-molecule structures, including ACA (CID: 41774), CGA (CID: 1794427), p-CA (CID: 637542), PC-B1 (CID: 11250133), and RUT (CID: 5280805), were retrieved from the PubChem database (http://pubchem.ncbi.nlm.nih.gov/, accessed on 12 April 2024), after which these compounds were hydrogenated (AutoDock Vina software 1.5.7). After docking, the binding was visualized and analyzed using the PyMol software 2.6 (http://www.pymol.org/, accessed on 20 May 2024). Key parameters, such as the binding energy (from AutoDock Vina software 1.5.7) and binding sites (from https://plip-tool.biotec.tu-dresden.de, accessed on 21 May 2024), were obtained from the docking results.

### 2.8. Molecular Dynamics

The MD of the phenolics interacting with the α-amylase was determined using the Gromacs 2023 software. The α-amylase and the phenolics-α-amylase complex were placed in a dodecahedron-shaped water box. The Amber14sb force field was utilized, along with the TIP3P model for water molecules. Sodium and chloride ions were added to achieve a net system charge of zero. The simulated system was subjected to a two-step energy minimization process. The first step involved 10,000 iterations using the steepest descent method, while the second consisted of 5000 iterations using the conjugate gradient method. After energy optimization, NVT and NPT simulations were performed for 200 ps each. The NPT simulation was followed by a production run for 100 ns. The temperature control algorithm was used in V-rescale, while the pressure control algorithm was used in Parrinello–Rahman, maintaining a temperature of 310 K and a pressure of 1 bar. Every 10 ps, Gromacs correlation commands were utilized to analyze the simulation results, after which the conformation data was saved.

### 2.9. Statistical Analysis

All results were presented as mean ± standard deviation based on three measurements. The differences between the groups were assessed via one-way analysis of variance (ANOVA) using IBM SPSS version 27.0.1 (SPSS Inc., Chicago, IL, USA). The statistical significance was denoted by a *p*-value below 0.05.

## 3. Results

### 3.1. Quantitative Analysis of SP

HPLC-MS/MS analysis identified 24 phenolic compounds in the SP extracts (Table S1). The main constituents comprised CGA (47.28%), p-CA (13.11%), peonidin 3-O-glucoside (7.69%), PC-B1 (3.54%), and RUT (2.81%). Peonidin 3-O-glucoside, an anthocyanin analog, is susceptible to degradation in neutral pH conditions [15]. Furthermore, the thermodynamic data suggest that its degradation rate markedly increases at elevated temperatures [16]. Consequently, follow-up experiments focused on the other four high-abundance components to investigate their interactions with α-amylase.

### 3.2. Inhibition Assays of the α-Amylase

**Figure 1:**
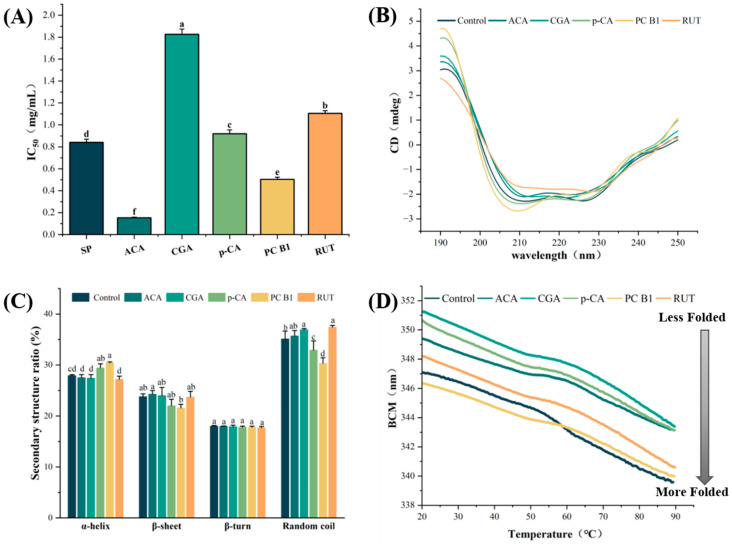
(**A**) The IC_50_ values after α-amylase exposure to phenolics and ACA. (**B**) The CD spectra after α-amylase exposure to phenolics and ACA. (**C**) The proportions of the secondary α-amylase structures containing phenolics and ACA. (**D**) The effect of phenolics and ACA on α-amylase stability at the same mass concentration (0.2 mg/mL). The values are expressed as means ± SD. Different lowercase letters denote significant differences among the groups (*p* < 0.05).

The inhibitory impact of SP and its main components on the α-amylase was investigated using ACA as a positive control (Figure 1A); the IC_50_ of SP on α-amylase was 0.841 ± 0.029 mg/mL. Of the four primary components, PC-B1 exhibited the most potent inhibitory effect on the α-amylase (IC_50_ = 0.504 ± 0.019 mg/mL). PC-B1 is a dimer of two flavan-3-ol monomers linked via a C4→C8 bond condensation. Previous research demonstrated that the phenolic hydroxyl group in the flavan-3-ol monomer can form hydrogen bonds with the active enzyme site to inhibit enzyme activity [17]. Another study found that proanthocyanidin-rich peel extracts significantly inhibited α-amylase. The inhibitory potential increased with the average degree of proanthocyanidin polymerization [18]. Although this indicated weaker inhibition than the positive ACA control, they still demonstrated some inhibitory potential.

RUT (IC_50_ = 1.105 ± 0.025 mg/mL) and CGA (IC_50_ = 1.826 ± 0.047 mg/mL) displayed a significantly lower inhibitory activity toward α-amylase than ACA (*p* < 0.05). This may be attributed to the spatial site-blocking effect induced by the RUT glycosylation modification and a weakened active enzyme binding site due to the caffeoyl moiety in CGA. This was consistent with the findings of Song et al. [19], who revealed that the caffeoyl moiety significantly reduced the inhibitory effect of CGA on α-amylase.

### 3.3. CD Spectral Analysis

CD spectroscopy is an effective technique for examining secondary structural changes in protein molecules [20]. As shown in Figure 1B, the π → π* and n → π* leap transitions produced two negative characteristic peaks at 208 nm and 226 nm, which were typical of the α-helix conformation [21]. As illustrated in Figure 1C, the addition of PC-B1 or p-CA elevated the α-helix content from 27.9 ± 0.20% to 30.4 ± 0.21% and 29.4 ± 0.83%, respectively, while reducing the β-folding content from 23.8 ± 0.56% to 21.6 ± 0.70% and 22.0 ± 1.29%, respectively. The results indicated that PC-B1 and p-CA altered the secondary enzyme structure by raising the α-helix content and lowering the β-sheet levels, which contributed to their inhibitory effect. However, RUT and CGA decreased the negative amylase peaks at 208 nm and 226 nm, which increased the random coil content from 35.1 ± 1.55% to 37.4 ± 0.36% and 36.9 ± 0.26%, respectively. A higher random coil level increased the α-amylase conformational flexibility, which undermined secondary-structure stability and disrupted tight binding between the substrate and the active enzyme site. Analysis of the IC_50_ values indicated that the α-helical conformation played a pivotal role in modulating the amylase inhibitory activity. Furthermore, Zheng et al. reported that amylase inhibitory activity was closely related to the α-helical conformation [22]. However, this study did not explore the potential role of random coils in enzyme activity.

### 3.4. Fluorescence Quenching Analysis

α-Amylase contains several aromatic amino acid residues, including Trp58, Trp59, and Tyr62, which exhibit stacking interactions and confer fluorescence at the entrance of the active site [8]. Changes in the microenvironments of these residues can be tracked by measuring the fluorescence intensity fluctuations and maximum emission wavelengths [23].

**Figure 2:**
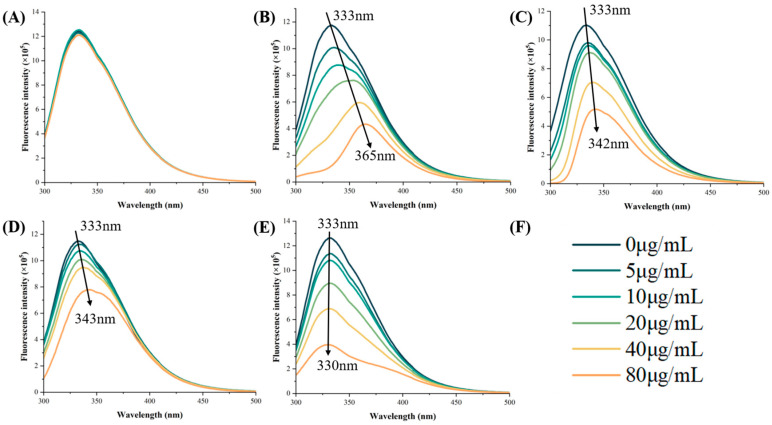
The fluorescence spectra of the α-amylase with (**A**) ACA, (**B**) CGA, (**C**) p-CA, (**D**) PC-B1, and (**E**) RUT. (**F**) the concentration of phenolic substances..

In the present study, although ACA effectively inhibited the α-amylase activity (Figure 2A), it did not significantly affect its intrinsic fluorescence. This could be due to the absence of an aromatic ring in the ACA molecular framework, which impeded π–π coupling with aromatic residues in the active site. Consequently, ACA failed to alter the microenvironments around Trp and Tyr. This result was consistent with the conclusion of Martinez-Gonzalez, who found that the ACA did not significantly affect the intrinsic fluorescence of pancreatic α-amylase [24]. Contrarily, CGA exhibited a significant fluorescence quenching ability (Figure 2B), possibly involving two action mechanisms. First, hydrophobicity enabled entry into the hydrophobic lumen of the enzyme. Second, the formation of specific hydrophobic interactions with Tyr62 directly affected the microenvironment of the active site. This supported the suggestion of Song et al. that there is not necessarily a positive correlation between inhibitory activity and the fluorescence quenching effect [19].

Further analysis indicated that, except for ACA, all the tested phenolic compounds showed concentration-dependent fluorescence quenching at the characteristic peak at 332 nm. Notably, CGA, p-CA, and PC-B1 produced distinct fluorescence redshifts (Figure 2B–D), implying that the aromatic residues migrated from the hydrophobic core into a more polar environment. Conversely, RUT caused a blueshift, likely burying the Trp residues deeper within the enzyme due to hydrogen bonding or hydrophobic effects, consequently increasing the local hydrophobicity [12]. Both redshifts (as seen in prunus leaf proanthocyanidin BLPs [13]) and blueshifts (as in chrysin or quercetin [12]) significantly inhibited enzyme activity. This suggests that the conformational change itself, potentially due to the SP action mechanism, may be more critical than the specific direction of the shift.

### 3.5. Analysis of the α-Amylase Stability

α-Amylase is a single polypeptide consisting of approximately 475 amino acid residues. It hydrolyzes starch and polysaccharides into smaller molecules such as glucose and maltose [25]. Since its structure and stability are pivotal for its enzymatic functionality, this study evaluated the thermal stability of α-amylase by monitoring the endogenous fluorescence changes during protein melting under UV excitation [26]. The fluorescence barycentric mean (BCM) can quantitatively characterize the dynamic folding process of proteins and provide a reliable system for evaluating enzyme thermal stability. Various 0.2 mg/mL phenolic compounds were introduced into the reaction, and the changes in the α-amylase fluorescence during heating were observed to investigate the potential impact on enzyme stability.

As shown in Figure 1D, the BCM shifted from 347.1 nm to shorter wavelengths at higher temperatures, indicating tighter folding coupled with denaturation and aggregation. The addition of CGA or p-CA significantly increased the BCM to 351.2 nm and 350.6 nm, respectively, suggesting more substantial Trp exposure to the aqueous environment, which resulted in a looser protein conformation and influenced the denaturation process. Below 55 °C, all the phenolic compounds, except for PC-B1, elevated the BCM values and decreased the structural density. However, above 60 °C, all the tested compounds further reduced α-amylase folding, exacerbating its unfolding and denaturation. PC-B1 yielded the densest α-amylase conformation, aligning with its more potent inhibitory activity, which was likely due to restricted substrate entry and binding. Contrarily, CGA caused less dense α-amylase folding, resulting in the loosest structure configuration. These findings were consistent with the CD results.

### 3.6. Molecular Docking Analysis

Molecular docking was performed to characterize the binding sites, binding energies, and interactions between the amylase and the main active SP components, based on the observed inhibitory effects of the phenolic compounds on the α-amylase activity and enzymatic conformational changes. As shown in Table 1, the binding energies of ACA (−6.9 kcal/mol), CGA (−7.6 kcal/mol), p-CA (−6.2 kcal/mol), PC-B1 (−7.8 kcal/mol), and RUT (−8.4 kcal/mol) with α-amylase were substantially lower than −4.6 kcal/mol, indicating that each compound formed stable complexes with the enzyme [27]. Despite displaying lower binding energies than the ACA positive control, PC-B1 and CGA exhibited weaker inhibitory abilities. This suggests that the inhibitory capacity of the enzyme is not governed by binding energy alone. Instead, factors such as the structural characteristics of the enzyme, conformational changes, hydrogen bonding, and various interaction forces may also play a critical role in regulating inhibitory activity. This was consistent with the findings of Khan et al. [28], who emphasized the importance of n-π interactions in enhancing enzyme inhibitory efficiency.

**Figure 3:**
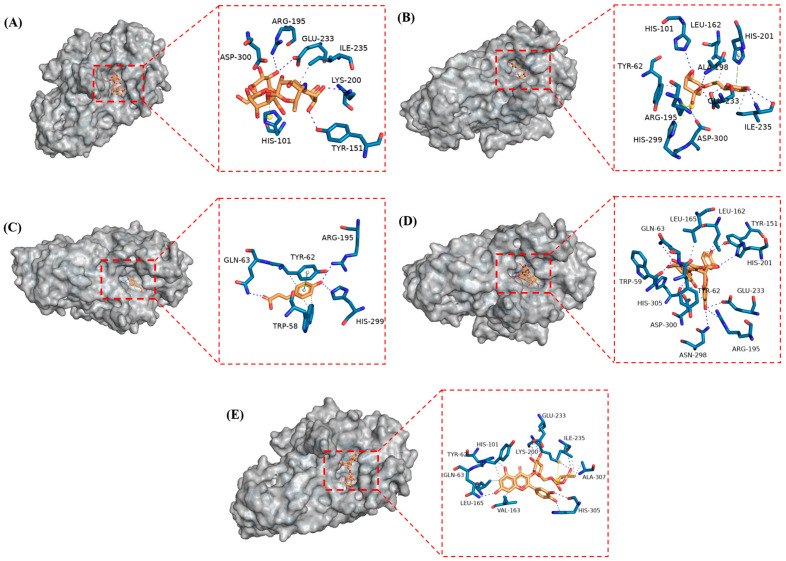
The molecular docking visualization of α-amylase binding with (**A**) ACA, (**B**) CGA, (**C**) p-CA, (**D**) PC-B1, and (**E**) RUT. Note: grey dotted lines represent hydrophobic interactions, blue solid lines represent hydrogen bonds, green dotted lines represent π-π stacking, and yellow dotted lines represent salt bridges.

The docking results indicated that all four phenolic compounds, as well as ACA, occupied the active amylase pocket and exerted an inhibitory effect by interacting with key residues such as Asp197, Glu233, and Asp300 (Figure 3). Glu233 formed two hydrogen bonds with CGA and RUT, and one with PC-B1 and ACA. Ile235 established hydrophobic interactions and hydrogen bonds with CGA and RUT, while Asp300 formed two hydrogen bonds with ACA and one with CGA. Gln63 established one hydrogen bond with the other phenolics, except CGA. These findings suggest that Tyr62, Glu233, Ile235, Gln63, and Asp300 are critical sites for phenolic-induced α-amylase inhibition.

Hydrogen bonding and hydrophobic interactions commonly underpin polyphenol–α-amylase binding [29]. As shown in Table 1, PC-B1 established nine hydrogen bonds (with Trp59, Gln63, Tyr151, Arg195, His201, Glu233, Asn298, and His305) and four hydrophobic interactions (with Tyr62, Leu162, Leu165, and Asp300). These contacts likely enhanced the inhibitory potency of PC-B1. Furthermore, research indicated that the synergistic effect of hydrophobic interactions and hydrogen bonding was essential for enzyme functionality [30]. During fluorescence quenching, the small molecule ligands bound to proteins via hydrophobic interactions can shift the polarity around aromatic residues to alter the fluorescence intensity. As shown in Table 1, Tyr62 hydrophobically interacted with four phenolic compounds, excluding ACA, possibly explaining why these four phenolics exhibited fluorescence quenching. Furthermore, RUT exhibited more significant hydrophobic interaction with the amylase, potentially contributing to the blueshift observed in its fluorescence spectrum.

### 3.7. Molecular Dynamics Analysis

While molecular docking techniques reveal static snapshots of the phenolics bound within the amylase active site, they offer limited insight into the dynamic nature of protein–ligand interactions [31]. Therefore, this study conducted 100 ns MD simulations using ACA and four phenolic compounds to predict the protein–ligand binding kinetics and stability [32]. Next, trajectory analyses were performed to assess these complexes’ stability, volatility, density, and solvation degree. This was achieved by evaluating the root mean square deviation (RMSD), root mean square fluctuation (RMSF), radius of gyration (Rg), and solvent-accessible surface area (SASA) of the protein receptors.

**Figure 4:**
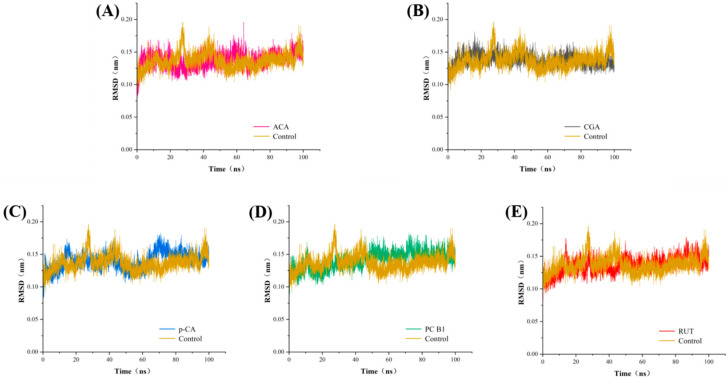
The RMSD simulations of the α-amylase with (**A**) ACA, (**B**) CGA, (**C**) p-CA, (**D**) PC-B1, and (**E**) RUT.

The RMSD quantifies the extent of protein conformation deviation from its initial structure at a given time and is often used to rapidly assess whether a simulation has achieved equilibrium [33]. Generally, if the RMSD is maintained within a small fluctuation range, the system is considered to have reached equilibrium and can undergo further analysis. As illustrated in Figure 4, the backbone RMSD of the α-amylase experienced considerable fluctuations (0.07–0.20 nm) from 0 ns to 40 ns, before stabilizing between 40 ns and 100 ns at around 0.12 nm. The backbone RMSD of the α-amylase-ACA complex converged near 0.12 nm at 65 ns to 100 ns. The α-amylase-CGA complex reached an equilibrium of approximately 0.13 nm between 55 ns and 100 ns. Conversely, the α-amylase–RUT and α-amylase–PC-B1 complexes stabilized at about 0.14 nm and 0.15 nm, respectively, between 50 ns and 100 ns. The backbone RMSD of the α-amylase-p-CA complex remained relatively stable from 70 ns to 100 ns, equilibrating around 0.15 nm. Overall, the RMSD values of these five systems indicated that they essentially reached equilibrium after 70 ns, confirming that all simulated systems reached a steady state. Consequently, the kinetic simulations are considered reliable for subsequent kinetic simulation analyses [20].

**Figure 5:**
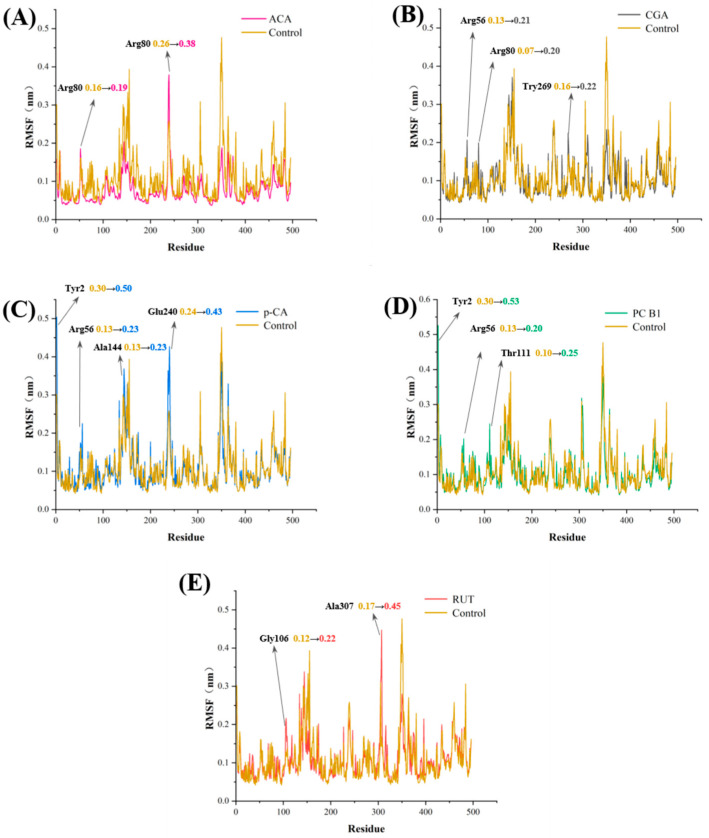
The RMSF simulations of the α-amylase with (**A**) ACA, (**B**) CGA, (**C**) p-CA, (**D**) PC-B1, and (**E**) RUT.

The RMSF is used to assess the levels of amino acid residue fluctuations during protein–ligand interactions, providing insight into protein flexibility [34]. Higher RMSF values indicate more significant amino acid residue fluctuation. Figure 5 shows the amino acid residue fluctuation in the α-amylase after binding with various phenolic substances. A few amino acid residues in the five complexes displayed slightly higher RMSF values than those observed in the unbound α-amylase. This suggested that adding phenolic substances to the α-amylase enhanced the movement of certain amino acid backbone residues.

However, the RMSFs of the key amino acid residues (Tyr62, Glu233, Ile235, Gln63, and Asp300) remained within a narrow range. This indicated that these five substances effectively limited residue fluctuation throughout the simulation, consequently affecting the enzyme-substrate binding and leading to inhibitory activity. Additionally, most amino acid residues showed consistently low fluctuations during the simulation, suggesting that the five complexes remained stable throughout the MD simulation [35]. The binding energy values obtained via molecular docking support the notion that these five substances can form stable complexes with α-amylase.

**Figure 6:**
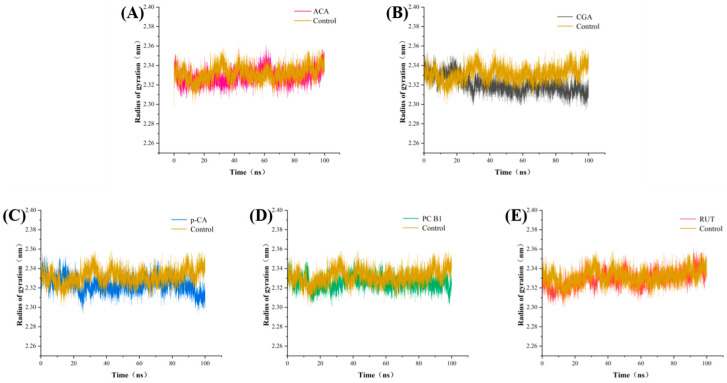
The Rg simulations of the α-amylase with (**A**) ACA, (**B**) CGA, (**C**) p-CA, (**D**) PC-B1, and (**E**) RUT.

The Rg of the proteins was measured to assess the density of the complexes. A larger Rg indicates a more significant structural change, which may be associated with partial unfolding or deformation [36]. As shown in Table S2, after the addition of the five substances, the complexes exhibited lower Rg values than α-amylase alone. This indicated that the binding of these five phenolic compounds enhanced the density of the amylase molecular structure. After 50 ns, all the curves remained stable (Figure 6), indicating that the complexes formed between these four phenolics and amylase exhibited relatively stable folded conformations [37]. These results suggest that phenolic binding increases the density of the protein structure, consequently enhancing its overall stability.

**Figure 7:**
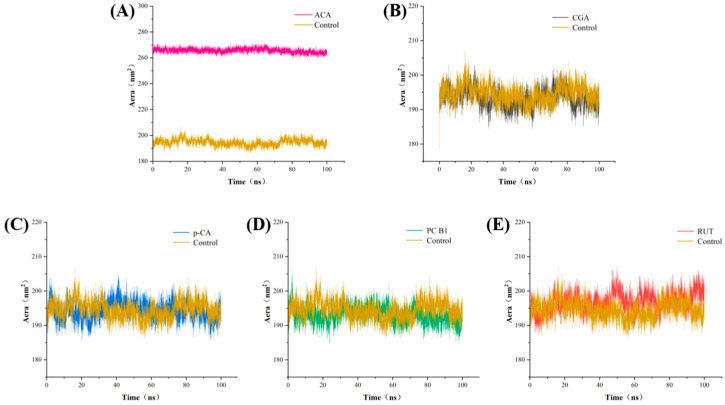
The SASA simulations of the α-amylase with (**A**) ACA, (**B**) CGA, (**C**) p-CA, (**D**) PC-B1, and (**E**) RUT.

The SASA value is used to characterize the portion of amylase that interacts with the solvent (water). A larger SASA value indicates a greater contact area between amylase and aqueous solution [37]. As shown in Figure 7 and Table S2, the SASA value for the α-amylase-ACA complex system was 266 nm^2^, significantly higher than that of α-amylase (195 nm^2^). This was likely due to the binding between ACA and α-amylase, which significantly changed the enzyme conformation, possibly reducing enzyme stability and surface hydrophobicity, ultimately enhancing its inhibitory capacity [38]. The SASA value of the RUT-amylase complex system increased to 197 nm^2^, suggesting that it altered the local structural folding of the protein. The CD assays further corroborated these structural changes, which revealed a significant increase in the random coil structures in the complex system. The phenolic–amylase complex system displayed altered SASA values, further suggesting that phenolic compounds affect the structural stability of α-amylase by modulating the distribution of hydrophobic regions on its surface [39].

In conclusion, the MD simulations of the α-amylase with four phenolics and ACA showed that these substances bound to the active enzyme site, forming stable complexes and inducing structural changes in the amylase. The complex formed by RUT, ACA, and α-amylase showed lower binding stability than those established by the other phenolics. Moreover, the key amino acid residues (Tyr62, Glu233, Ile235, Gln63, and Asp300) exhibited lower fluctuation throughout the simulation, stabilizing the enzyme conformation and playing a pivotal role during the binding process. These findings are consistent with the molecular docking results.

## 4. Conclusions

This study systematically investigates the inhibitory effect of SP on α-amylase and elucidates its action mechanism by integrating computer simulations with protein conformation analysis. The findings indicate that SP is a promising α-amylase inhibitor, with the potential to lower blood glucose levels. The results reveal that SP exerts an inhibitory effect by altering the secondary enzyme structure, which affects its overall stability and the microenvironment of the aromatic amino acid residues. The mechanism analysis shows that hydrogen bonding and hydrophobic interactions play crucial roles in binding phenolics and α-amylase. The molecular docking results indicate that Tyr62, Glu233, Ile235, Gln63, and Asp300 may be the key residues involved in SP and α-amylase binding. This finding is consistent with the residue fluctuations observed during MD.

Proanthocyanidin B1 exerts enzyme inhibition by occupying the binding site, rigidifying the catalytic structure, and destroying the microenvironment of the active center. It exhibits better inhibition than SP extracts with equivalent polyphenol levels. This suggests that PC-B1 may be the key active ingredient in SP, playing a critical role in its hypoglycemic functionality. However, the oral bioavailability of PC-B1 is significantly limited due to its low absorption in the gastrointestinal tract, poor chemical stability, and complex metabolic processes. To address these challenges, future research should concentrate on quantitatively analyzing the kinetics of its metabolites and optimizing delivery systems, such as nanocarriers and phospholipid complex technologies. These efforts aim to enhance the therapeutic efficacy and clinical potential of PC-B1.

This study provides a theoretical basis for SP as a potential natural α-amylase inhibitor, highlighting its applications in functional foods and blood glucose management. However, the current study provides only a preliminary investigation of the enzyme-inhibiting effects of the main components in polyphenol extract. Future research should explore the synergistic and antagonistic interactions between the trace components and the main components to better understand the overall inhibitory mechanisms of these polyphenol mixtures. Additionally, further verification of their bioavailability in vivo and whether they produce similar effects is needed to evaluate their practical applications in dietary interventions.

## Figures and Tables

**Table 1:** The binding sites between the different substances and α-amylase.

Substance	Binding Energy (kcal/mol)	Hydrophobic Interaction		Hydrogen Bond		π-π Stacking		Salt Bridge	
ACA	−6.9	Ile235	1	Tyr151, Arg195, Lys200, Glu233, Asp300	6	ACA	−6.9	Ile235	1
CGA	−7.6	Tyr62, Leu162, Ile235	3	His101, Ala198, Glu233, Ile235, Asp300	7	CGA	−7.6	Tyr62, Leu162, Ile235	3
p-CA	−6.2	Trp58, Tyr62	3	Gln63, Arg195, His299	3	p-CA	−6.2	Trp58, Tyr62	3
PC-B1	−7.8	Tyr62, Leu162, Leu165, Asp300	4	Trp59, Gln63, Tyr151, Arg195, His201, Glu233, Asn298, His305	9	PC-B1	−7.8	Tyr62, Leu162, Leu165, Asp300	4
RUT	−8.4	Tyr62, Val163, Leu165, Ile235, Ala307	5	Gln63, His101, Lys200, Glu233, Ile235, His305	8	RUT	−8.4	Tyr62, Val163, Leu165, Ile235, Ala307	5

Note: Red indicates the formation of two forces.

## Data Availability

The original contributions presented in the study are included in the article/Supplementary Materials. Further inquiries can be directed to the corresponding author.

## Supplementary Materials

The following supporting information can be downloaded at: https://www.mdpi.com/article/10.3390/foods14132174/s1, Table S1: analysis of major phenolics in sugarcane polyphenols; Table S2: average Rg and SASA values of different substances with α-amylase.

## Abbreviations

The following abbreviations are used in this manuscript:

SP	Sugarcane Polyohenol
ACA	Acarbose
CGA	Chlorogenic acid
p-CA	p-Coumaric acid
PC-B1	Proanthocyanidin-B1
RUT	Rutin
CD	Circular Dichroism
MD	Molecular dynamics
BCM	Barycentric mean
HPLC-MS/MS	High-performance Liquid Chromatography–Tandem Mass Spectrometry
