# Supplementary material for: Effect of Sugarcane Polyphenol Extract on α-Amylase Inhibition and Mechanism Exploration
Source: Foods. 2025 Jun 21;14(13):2174. doi: 10.3390/foods14132174 (PMC12248944; doi:10.3390/foods14132174)
Supplement: Supplementary file 1 [file foods-14-02174-s001.zip › foods-3692846-supplementary.pdf]

Supporting Information

Table S1. Analysis of major phenolics in sugarcane polyphenols.

No.	Title	Formula	RT (min)	Precursor m/z	Adduct
1	chlorogenic acid	$C_{16}H_{18}O_9$	5.360633	353.0861	[M-H]-
2	p-coumaric acid	$C_9H_8O_3$	6.955217	163.0408	[M-H]-
3	peonidin 3-O-glucoside	$C_{22}H_{23}O_{11}$	6.587234	462.1119	[M-H]-
4	procyanidin B1	$C_{30}H_{26}O_{12}$	6.831717	577.1532	[M-H]-
5	procyanidins	$C_{30}H_{26}O_{13}$	5.973433	593.146	[M-H]-
6	rutin	$C_{27}H_{30}O_{16}$	5.932117	609.1415	[M-H]-
7	hesperetin	$C_{16}H_{14}O_6$	1.722833	301.0907	[M-H]-
8	luteolin 7-O-glucoside	$C_{21}H_{20}O_{11}$	6.628067	447.0906	[M-H]-
9	isorhamnetin	$C_{16}H_{12}O_7$	1.805167	315.0711	[M-H]-
10	kaempferol	$C_{15}H_{10}O_6$	8.980933	285.0381	[M-H]-
11	ferulic acid	$C_{10}H_{10}O_4$	7.365533	193.0513	[M-H]-
12	isoquercetin	$C_{21}H_{20}O_{12}$	7.3192	481.1147	[M+H]+
13	apigenin 7-O-glucoside	$C_{21}H_{20}O_{10}$	6.996383	431.0953	[M-H]-
14	genistein	$C_{15}H_{10}O_5$	7.443017	271.0618	[M+H]+
15	gallic acid 4-O-glucoside	$C_{13}H_{16}O_{10}$	10.25503	331.2386	[M-H]-
16	Pinocembrin	$C_{15}H_{12}O_4$	20.1865	255.2318	[M-H]-
17	p-coumaroyl malic acid	$C_{13}H_{12}O_7$	19.08573	279.2315	[M-H]-
18	cinnamic acid	$C_9H_8O_2$	5.05365	149.0594	[M+H]+
19	formononetin	$C_{16}H_{12}O_4$	1.315017	267.0701	[M-H]-
20	naringenin	$C_{15}H_{12}O_5$	1.640833	271.0847	[M-H]-
21	biochanin A	$C_{16}H_{12}O_5$	6.415583	285.0748	[M+H]+
22	catechin gallate	$C_{22}H_{18}O_{10}$	6.044933	443.0983	[M+H]+
23	cyanidin	$C_{15}H_{11}O_6$	11.04382	288.2899	[M+H]+
24	phloretin	$C_{15}H_{14}O_5$	1.346517	275.0751	[M+H]+

Table S2. Average Rg and SASA values of different substances with α-amylase.

Title	Rg (nm)	SASA (nm^2)
α-amylase(control)	2.331834	195
acarbose	2.328683	266
chlorogenic acid	2.320616	193
p-coumaric acid	2.323548	194
procyanidin B1	2.325347	193
rutin	2.329259	197
